# Research on the damage characteristics of rock masses based on double guide-hole blasting under high in-situ stress

**DOI:** 10.1038/s41598-023-44985-9

**Published:** 2023-11-06

**Authors:** Ruizong Xu, Liangfu Xie, Long Ma, Benfeng Wang

**Affiliations:** 1 College of Civil Engineering and Architecture, Xinjiang University, Urumqi, 830017 China; 2 Xinjiang Civil Engineering Technology Research Center, Urumqi, 830017 China; 3 Xinjiang Academy of Architectural Science (Limited Liability Company), Urumqi, 83002 China; 4 Xinjiang Changji Vocational and Technical College, Changji, China

**Keywords:** Civil engineering, Engineering

## Abstract

In complex high in-situ stress conditions, how to achieve the ideal rock blasting effect through effective methods is often a difficult point of blasting operations. This paper analyzes the influence of guide holes on blasting effect by adding guide holes to the rock pre-treatment method. Based on the particle expansion method to carry out double-hole blasting experiments, the influence of the blasthole spacing and ground stress on the blasting effect is investigated from the levels of macroscopic cracking effect, microscopic particle contact and so on. The study shows that: (i) the setting of empty holes between the gun holes can enhance the crack penetration effect, and the penetration effect is more obvious when the distance between the gun holes and the empty holes is less than 2.5 times the radius of the crushed zone. (ii) At the level of contact force chain, when the distance between blastholes and empty holes is less than 2.5 times the radius of the crushing zone. The compressive stress in the direction perpendicular to the direction of the blasthole line inhibits crack development, and the tensile stress in the direction parallel to the blasthole line promotes crack development. The main stress direction is perpendicular to the direction of the blasthole line. (iii) As the distance between the blastholes increases, the effect of crack suppression by stresses in the vertical direction decreases, and the main force direction is parallel to the direction of the blasthole line.

## Introduction

Blasting is an effective method of rock breaking in deep engineering activities such as excavation of tunnels and exploitation of underground resources, and the ideal section of blasting excavation is a topic of concern for blasting operations, which has a certain impact on the subsequent engineering activities. Therefore, it is significant to achieve the ideal effect and increase the controllability through the improvement of some methods. For this reason many scholars have done more research, some scholars start from the explosives improvement^1,2^, some analyze from the level of blasting environment^3^, rock pre-treatment^4^ and so on. Due to the extensive research on explosives improvement and the complexity of the natural environment, the use of rock pre-treatment, such as the use of guide holes to induce rock blasting appears to be more reasonable.

Due to the exhaustion of resources in the shallow part of the earth and the rapid development of underground engineering, deep earth blasting has been widely popularized, and in-situ stress is one of the main factors affecting blasting, and scholars have studied more at the level of in-situ stress. It is found that the ground force has a large influence on the propagation of explosive stress^5,6^; high ground stress significantly affects the degree of rock fragmentation^7^; and the initial stress near the blast hole will have an effect on early cracking^8^. At the practical application level, Yuan et al.^9^ investigated the damage mechanism of surrounding rock when the in-situ stress is coupled with the blasting load. Cui et al.^10–12^ analyzed the blasting law of soft and hard rock formations under high in-situ stress at the level of the contact synergies, energy. Yang et al.^13^ investigated the effect of in-situ stress on the attenuation of the blast energy; and found that the stress redistribution is the most important factor that leads to the damage of rock mass after the explosion of explosives^14^. Zhao et al.^15^ found that the risk zone caused by blasting was concentrated in the downstream sidewall of underground caves. Scholars have studied more at the level of the effect of in-situ stress on blasting, but the effect of in-situ stress on blasting of rock bodies containing guide holes has been rarely analyzed.

Guide hole blasting is a blasting method to pre-treat the rock mass. Guide holes can induce crack expansion and control the effect of blasting, for which many scholars have conducted research. A large number of experiments in previous studies used Plexiglas (PMMA) to study the effect of guide holes on crack guidance, and it was found that when the distance between guide holes and empty holes was less than 1 m, the crack penetration effect was better^16–20^. While the distance between guide holes and empty holes was less than 1 m in soft rock conditions, the guide holes guided the cracks better^21,22^. Therefore, comparing to the conclusions of guide hole blasting in soft rock, PMMA was used to study the effect of Guide hole blasting in soft rock is more accurate. And in hard rock conditions. Only a small number of scholars based on the finite element method and experimental method found that the crack penetration effect is good when the distance between the gun hole and the air hole is 1.2 m and 1.6 m^23,24^. In this paper, the discrete element method is used for the first time in the hard rock blasting containing guide holes, which not only verifies the reasonableness of the conclusions obtained by the finite element method and experiments, but also further supplements the problem that the guide hole blasting has been slightly less researched in the hard rock layer.

Most of the research in the direction of guided hole blasting by scholars is aimed at soft rock, focusing on delay time, hole spacing and so on. There are fewer analyses on hard rock, ground stress, and micro contact level of rock body. This paper uses Particle Flow code (PFC), with the help of particle expansion discrete element blast simulation method^25^. Taking the hard rock with guide holes as the object of analysis, the blasting law under different hole spacing and ground stress is studied from the perspective of combining macro and microscopic.

## Numerical theoretical analysis

### Theoretical analysis of particle expansion method

**Figure 1:**
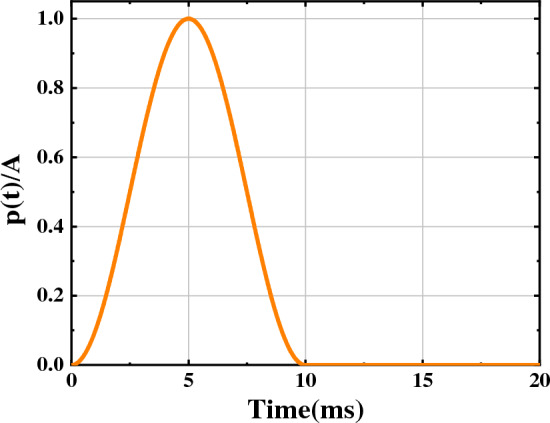
Time history curve of blasthole pressure.

The particle expansion method^25^ is a blasting simulation method based on the discrete element method. blasting load loading form is analogous to the form of a stress wave transmitted outward from the blast point. In this paper, the stress wave is abbreviated as a half-sine wave with the same ascending and descending segments, and according to Fig. 1, it is expressed as in Eq. (1)^25^:

1 $$ p_{{({\text{t}})}} = \frac{A}{2}(1.0 - \cos (2\pi ft)) $$

where $P_{{({\text{t}})}}$ is the burst pressure; *A* is the peak pressure of the explosive wall; $f$ is the half-sine wave frequency, the general explosion time is less than 1/*f* = 10 ms.

Since the charging method in this paper is uncoupled charging ($\xi > 1$), the solid theory analysis is based on uncoupled charging.

2 $$ \zeta = \frac{{d_{{\text{b}}} }}{{d_{{\text{e}}} }} $$

where $\xi$ is the uncoupling factor; *d*_b_ is the blasthole diameter; *d*_e_ is the explosive package diameter.

When the charge mode is uncoupled charge, and the explosion impact *p*_0_ reaches the blasthole wall, it recedes as:

3 $$ p_{0} = \frac{1}{8}\rho_{0} D^{2} \left( {\frac{{V_{{\text{c}}} }}{{V_{{\text{b}}} }}} \right)^{3} n $$

where $\rho_{0}$ is the density of explosives; *D* is the explosive burst speed; *V*_c_*,* *V*_b_ is the volume of the hole chamber and the volume of the package; n is the increase in the number of times, generally 8–11.

**Figure 2:**
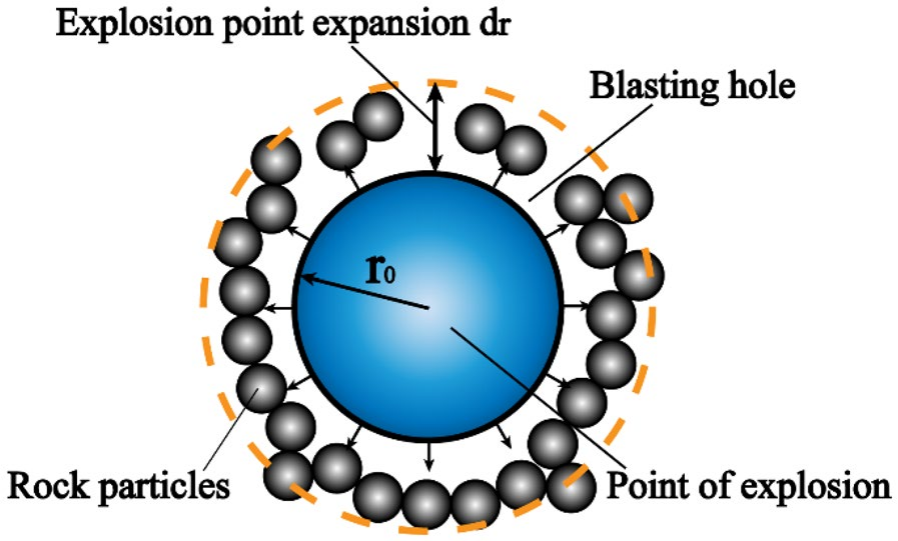
Diagram of blasting based on particle expansion method.

According to the principle of discrete element particle contact, the principle of particle expansion method^25^ is shown schematically in Fig. 2. When the explosion point expansion to the hole wall, the radial thrust $F = k_{{\text{n}}} d_{{\text{r}}} = 2\pi r_{0} p_{0}$ on the rock particles, the amount of expansion of the blast point *d*_r_ is:

4 $$ d_{{\text{r}}} = \frac{{2\pi r_{0} p_{0} }}{{k_{{\text{n}}} }} $$

5 $$ k_{{\text{n}}} = \frac{{2(r_{\max } + r_{\min } )\pi p_{0} }}{{(r_{\max } - r_{\min } )}} $$

where $r_{0}$ is the radius of the explosive package; $p_{0}$ is the hole wall pressure; $k_{{\text{n}}}$ is the contact stiffness; $r_{\max }$, $r_{\min }$ is the maximum and minimum radius of the blast point particles.

### Boundary conditions

To prevent the effect of reflected stress at the free surface on the blasting effect, it is crucial to utilize a transmissive flexible boundary. For this purpose scholars have proposed the dispersion effect (Chong Shi^25^) and flexible boundary conditions (Kouroussis^26^), which are used to achieve the effect of transmissive blast stress and better prevent the effect of reflected stress at the free surface on the blasting effect of the rock mass. The flexible boundary theory is as follows.

When the explosive explosion, the relationship between the boundary reaction force and the particle velocity is:

6 $$ F = - 2R\rho c\dot{u} $$

where *R* is the particle radius; $\rho$ is the medium density; *c* is the wave speed; and $\dot{u}$ is the particle velocity.

To prevent the dispersion effect on the blasting effect, it is crucial to use a correction factor to eliminate the dispersion effect. The corrected boundary reaction force is:

7 $$ F = \left\{ {\begin{array}{*{20}c} { - \zeta 2R\rho c_{{\text{p}}} \dot{u}_{{\text{n}}} } \\ { - \eta 2R\rho c_{{\text{s}}} \dot{u}_{{\text{n}}} } \\ \end{array} } \right. $$

where $\zeta$ and $\eta$ are the correction coefficients for the longitudinal and transverse wave dispersion effects; $c_{{\text{p}}}$ is the longitudinal wave velocity; $c_{{\text{s}}}$ is the transverse wave velocity; $\dot{u}_{{\text{n}}}$ is the particle normal motion velocity and $\dot{u}_{{\text{s}}}$ is the particle tangential motion velocity.

## Establishment of numerical model

### Specific experimental procedure

**Figure 3:**
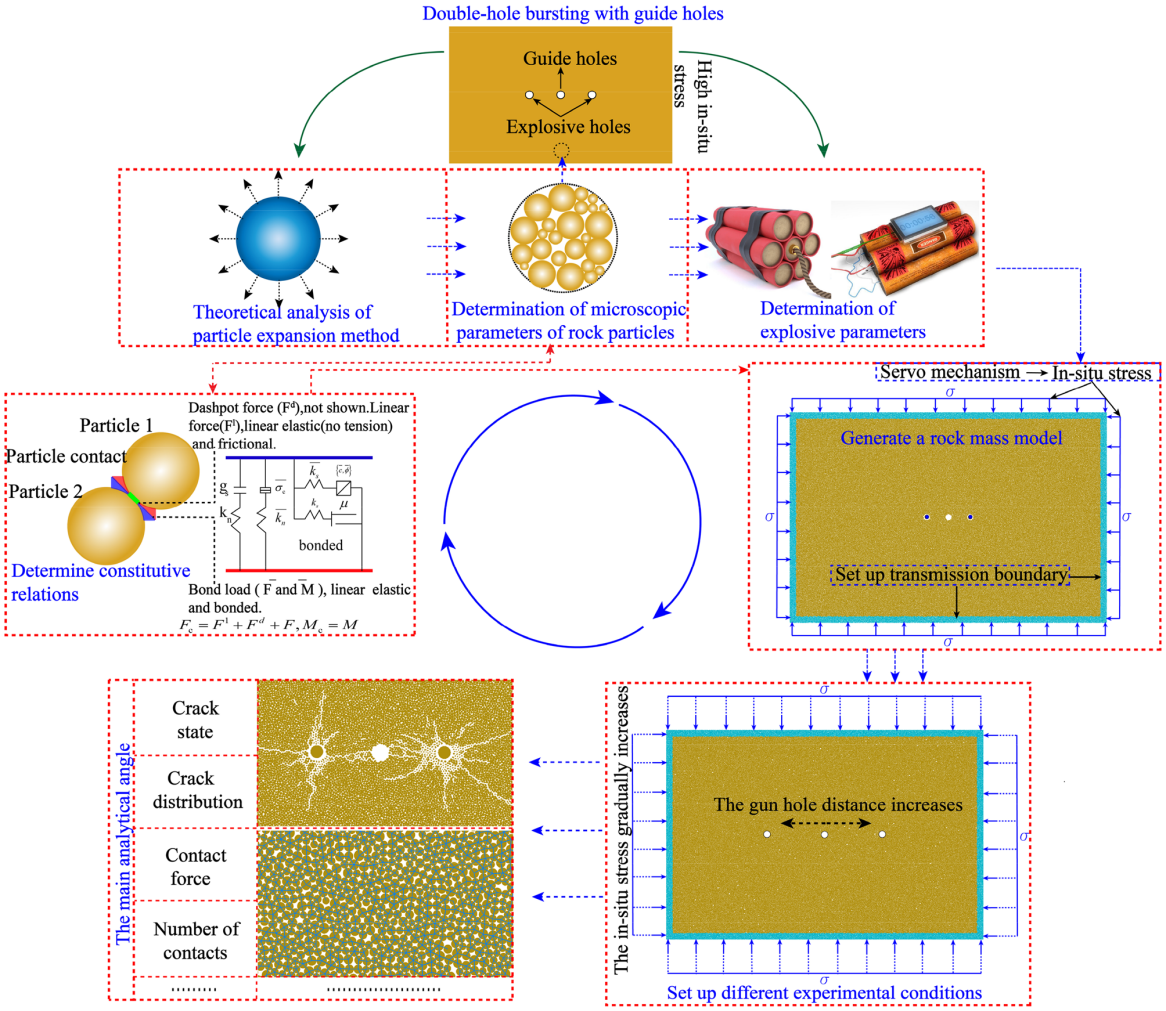
Flow chart of the experimental process.

In order to show the specific test process of this paper clearly, this chapter shows the overall test process of this paper in the form of test flow diagram, the article starts with the selection of fine view parameters, particle expansion loading theory, the determination of model and explosive parameters, followed by the determination of intrinsic model and boundary conditions, and comes up with different blasthole spacing and in-situ stress working conditions, the crack, the The variation law of contact force, contact number, etc. is shown in Fig. 3.

### Selection of microscopic parameters

**Figure 4:**
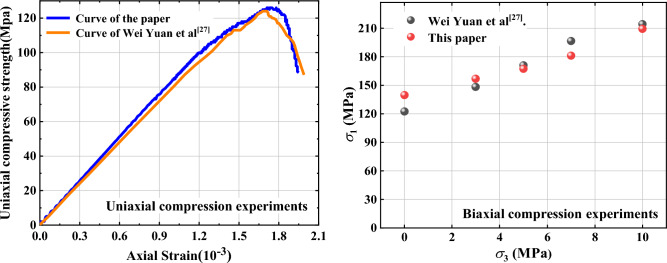
Schematic diagram of basic experimental validation.

In this paper, sandstone is used as the research object, because Yuan et al.^27^ conducted uniaxial and Brazilian splitting test on sandstone by test, and obtained the macroscopic parameters of sandstone, and then conducted uniaxial and biaxial test using PFC, and calibrated the fine view parameters using the macroscopic parameters of sandstone, solid the parameters of this paper were selected from those obtained by Yuan et al.^27^. To verify the rationality of the taken parameters, uniaxial and biaxial compression tests were performed using PFC, and the obtained test curves were basically consistent with the results obtained by Yuan under the same model size, particle size, and in-situ stress, as shown in Fig. 4, which verified the rationality of the microscopic parameters. The specific parameters are shown in Tables 1 and 2.

**Table 1:** Sandstone macro parameters^27^.

Elasticity modulus	Poisson's ration	Compression strength	Tensile strength
64.93 GPa	0.17	122.76 MPa	8.79 MPa

**Table 2:** Sandstone microscopic parameters^27^.

Parallel-bond group					Linear group		
Bond effective modulus	Bond tensile strength	Bond cohesion	Friction angle	Bond stiffness ratio	Effective modulus	Stiffness ratio	Friction coefficient
42 GPa	30 MPa	350 MPa	65°	1.0	51 GPa	1.0	1.0

### Validation of the extent of the crushing zone

The particle expansion method^25^ is a blasting simulation method based on discrete elements. In order to verify the rationality of the method, this paper compares the theoretical analytical solution of the crushing zone range with the numerical solution obtained from the simulation, and the specific demonstration process is as follows.

#### Crushing zone radius theoretical solution

At the level of rock blasting, the extent of the crushing zone is a key indicator of the response to the effect of rock breaking, for this reason some scholars have conducted a lot of research, scholars through a combination of theoretical derivation and practical experience in the form of semi-empirical formula for the radius of the crushing zone, this paper selected Kanchibotla et al.^28^ to obtain the semi-empirical formula for the crushing zone radius $r_{{\text{c}}}$ verification. The specific equation is as follows:

8 $$ r_{{\text{c}}} \, = \, r_{0} \sqrt {\frac{{p_{{\text{d}}} }}{{\sigma_{{\text{c}}} }}} $$

9 $$ p_{{\text{d}}} = \frac{1}{r + 1}\rho_{0} D^{2} $$

where $r_{0}$ is the blasthole radius; $p_{{\text{d}}}$ is the burst pressure; $\sigma_{{\text{c}}}$ is the unconfined compressive strength; $r$ is the multiparty index, the general case *r* = 3.

#### Numerical solution for the extent of the crushing area

**Figure 5:**
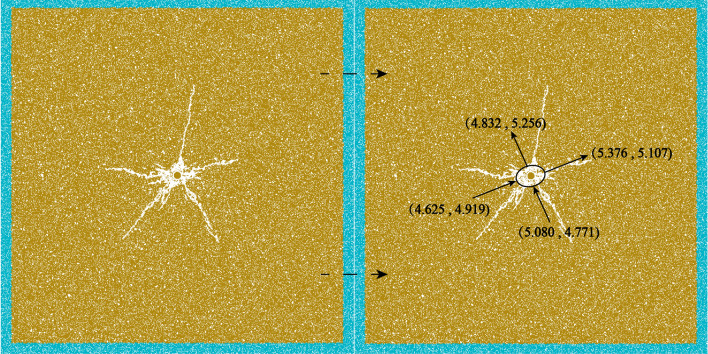
Schematic diagram of crushing zone verification.

For the crushing area obtained from numerical simulation is shown in Fig. 5, the model size is 10 m × 10 m, and other parameters are consistent with this paper. The crushing area exists in the form of a similar long ellipse, and in order to get the size of the radius of the crushing area, this paper calculates the area of the crushing area by the four vertices of the long ellipse, and the area of the crushing area can be obtained by analysis as about 1.328 m^2^ and the radius of the crushing area $r_{{\text{c}}}$ is about 0.650 m. $r_{{\text{c}}} /r_{0} = 4.55$, $r_{{\text{c}}} /r_{0}$ satisfies as 3–5 times the range^29^. Among them, the theoretical analytical solution was obtained $r_{{\text{c}}} /r_{0} = 4.28$. The error between the numerical solution and the theoretical solution is within a reasonable range, and the rationality of the particle expansion blasting simulation method can be verified.

### Establishment of discrete element model

**Figure 6:**
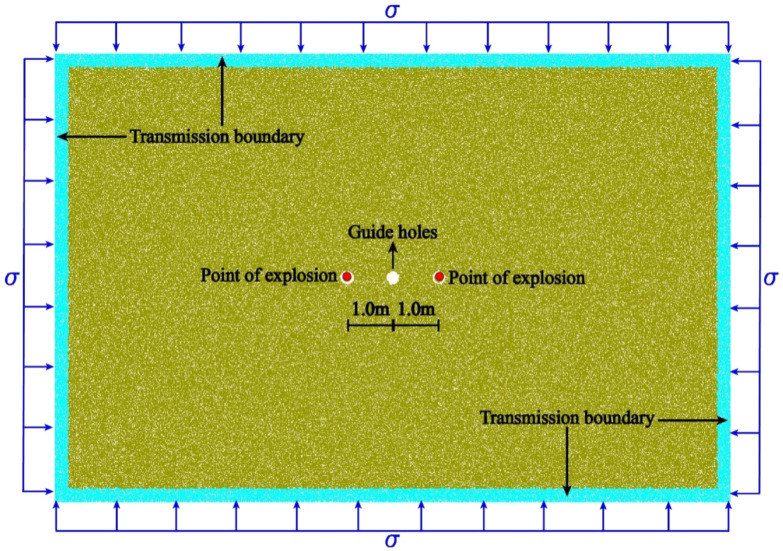
Schematic diagram of the blasting model.

In this paper, with the help of particle expansion blasting simulation method, sandstone is used as the research object to analyze the blasting damage characteristics of rock mass containing guard hole under high in-situ stress, and a total of 16 (4 × 4) working conditions are analyzed with the distance between in-situ stress and blasthole as variables, as shown in Table 3. the model is modeled by PFC2D for modeling, and the servo mechanism proposed by Cundall et al.^30^ was used to simulate the high in-situ stress environment. The overall size of the model is 15 m × 10 m, consisting of 99,133 particles with uniform particle size distribution in the radius range of 5–7.5 mm. the model boundary is set as a flexible transmissive boundary. The inter-particle contact is taken from the parallel bond model, which has a good bearing capacity for inter-particle forces and bending moments, and is reasonable for the simulation of rock particles. The blasthole spacing of L = 2 m was chosen for the model schematic, which is shown in Fig. 6.

**Table 3:** Experimental conditions.

Influencing conditions	I	II	III	IV
Gun hole distance (m)	2	3	4	5
In-situ stress (MPa)	5	20	40	60

Explosive load: the explosives selected in this paper are basically the same as the explosive properties selected by Yuan et al.^27^, where the package diameter $d_{{\text{e}}}$ = 10 cm, the explosive density $\rho$ = 1000 kg/m^3^, and the explosive burst speed *D* = 2700 m/s. The blasthole diameter *d* = 14 cm and the uncoupling factor $\xi$ = 1.4 in this paper.

## Analysis of extension characteristics of rock blasting crack

**Figure 7:**
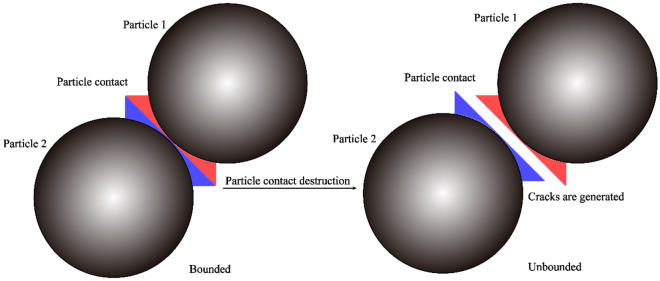
Schematic diagram of crack generation.

During blasting test, the development and final state of crack is a key indicator to reflect the blasting effect, and the more the number of cracks in the blasting disturbance range, the more serious the rock damage. In this paper, the development state of crack under different influencing factors is studied based on particle expansion method. For discrete element method crack generation principle, mainly related to the contact between the particles, when the force and moment between the particles withstand more than the limit state, the contact between the particles is destroyed, new crack generated, the specific crack generation schematic as shown in Fig. 7.

### Spatial development characteristics of crack

**Figure 8:**
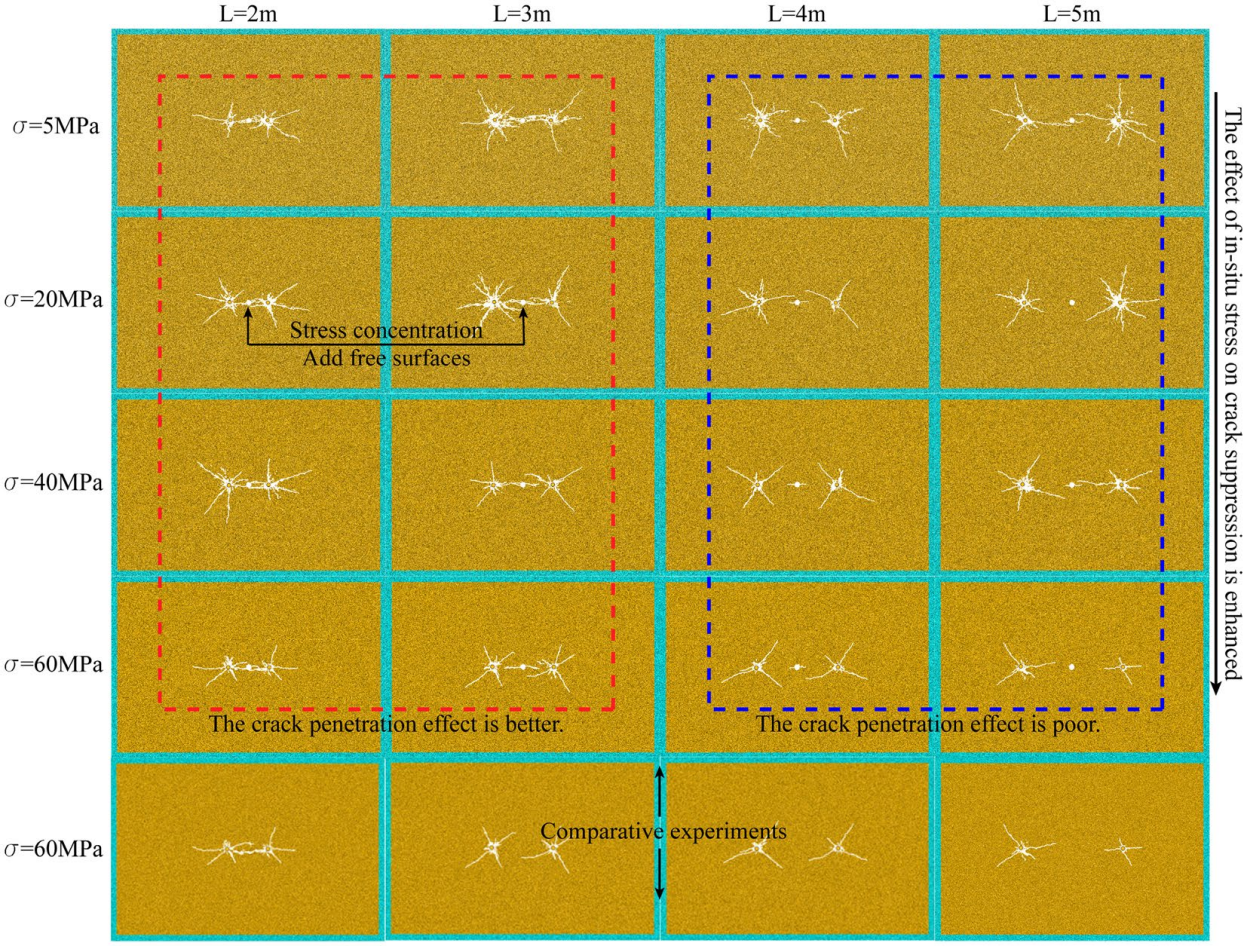
Crack grid diagram.

The double-hole blasting effect for each in-situ tress ($\sigma$) and different horizontal distance (L) working conditions of blasthole and blasthole (empty hole is located in the middle) are shown in Fig. 8.

As shown in Fig. 8, the distance between blasthole and blasthole (L) has a significant effect on the final blasting effect when the in-situ stress is 5 MPa (the distance between blasthole and hollow hole is half of the distance between blasthole). When L = 2 m and 3 m, it can be clearly seen that the presence of the hollow hole makes the area of the free surface increase, which also makes the stress wave reflect at the free surface and exhibits the stress concentration phenomenon caused by the hollow hole. The crack between the blasthole is caused to develop along the direction of the hollow hole and pass through the hollow hole, and the crack in the direction of the blasthole-hollow hole-blasthole link is denser and finally shows a well-developed through crack. with the increase of L, the crack around the blasthole decreases significantly when L = 4 m and L = 5 m. It indicates that when the distance between blasthole and blasthole is too large, the concentration of stress caused by the hollow hole will be relatively insignificant, leading to a substantial weakening of the penetration effect of the crack.

When the in-situ stress is 20 MPa, the overall pattern is the same as 5 MPa, but the penetration effect differs due to the increase of in-situ stress. At L = 2 m and 3 m, the penetration effect of crack is still better, but it is slightly weakened compared with the 5 MPa case. With the increase of L, the penetration effect of crack is still poor when L = 4 m and L = 5 m. When the in-situ stress is 40 MPa, the overall law is the same as 5 MPa and 20 MPa. At L = 2 m and 3 m, the penetration effect is better. With the increase of L, the penetration effect of crack is still poor when L = 4 m and L = 5 m.

When the in-situ stress is 60 MPa, the overall law is also basically consistent with the previous three working conditions. At L = 2 m, the penetration effect is more obvious, but weaker than the first three working conditions. When L = 3 m, it can be seen from Fig. 8 that the penetration crack between the left and right blasthole and the hollow hole have been broken, indicating that the suppression effect of 60 MPa in-situ stress is more obvious than that of 40 MPa. When L = 4 m and L = 5 m, the penetration effect of crack is still poor.

In order to reflect the effect of hollow hole can increase the penetration of crack under high in-situ stress, this paper takes 60 MPa as an example to do double-hole blasting experiment without hollow hole, and the results are shown in Fig. 8. At L = 2 m, the effect with and without hollow hole is basically the same due to the close distance of blasthole. When L = 3 m, the crack without hollow hole has basically no penetration effect, similar to two blasting without mutual interference, while when the hollow hole exists, the penetration effect of the crack is significantly enhanced than the case without hollow hole. When L = 4 m and 5 m, there is slightly crack or even no crack near the hollow hole, indicating that when the blasthole distance is larger, the role of the hollow hole in enhancing the crack penetration effect is seriously weakened or even disappeared.

In summary, when double-hole blasting, adding hollow holes between two blastholes can enhance the crack penetration effect between blastholes (this effect will become the hollow hole effect later in this paper). Taking the radius of the crushing zone of 2.3 chapters as the benchmark, the penetration effect is obvious when the distance between blasthole and hollow hole is less than 2.5 times the radius of the crushing zone, and the penetration effect is obviously weakened or even disappeared outside this range. By adjusting the distance between blasthole and hollow hole, the penetration effect can also be enhanced, so as to overcome the inhibiting effect of in-situ stress on crack to a certain extent.

Previous scholars based on finite element and experimental method. In the study of hard rock guide hole blasting, it is found that when the distance between the gun hole and the empty hole is 1.2 m and 1.6 m^23,24^, the crack penetration effect is good, which is mutually verified with the 1.5 m proposed in this paper.

### Statistics of the direction of crack

The information of crack, such as the number and direction, can reflect the damage degree of the rock body around the blasthole to some extent. In this paper, the information of cracks generated inside the rock mass after blasting is counted, and the statistical results are shown in the crack rose diagram.

**Figure 9:**
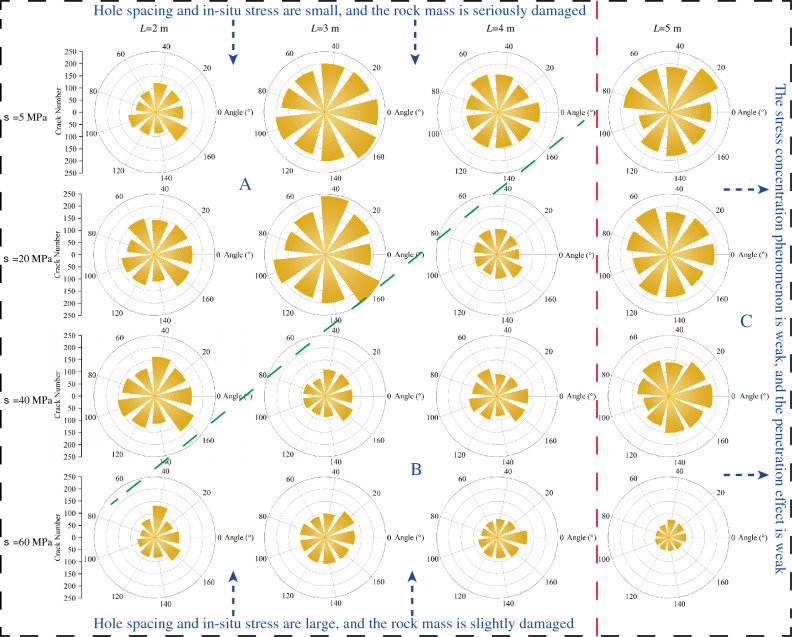
Crack rose diagram.

As shown in Fig. 9, the crack rose diagram is divided into three parts. According to the degree of influence of the interaction between blasthole, L > 4 m is divided into a part (i.e., the red dashed right part C area in the figure), and the change law of crack with in-situ stress in this part is basically consistent with the law of general single-hole pure hard rock, and the number of crack and the degree of rock fragmentation are decreasing and weakening obviously with the increase of in-situ stress. From the blasting crack network diagram and crack number diagram, when L = 5 m, the blasting process is closer to two independent single-hole blasting, and the interaction between blasthole and hollow hole effect is relatively weak. It indicates that when the distance between blasthole and blasthole is too large, even if the hollow holes are added between blasthole, it will not play an obvious stress concentration effect and thus will not enhance the crack penetration effect.

The L ≤ 4 m is divided into another part (i.e., in the left part of the red dashed line in the figure, the green dashed line upper part A and the green dashed line lower part B), in which the distance between blastholes is smaller, the interactions between blastholes and the effect of vacuoles are more obvious, and the relationship with in-situ stress is relatively complicated. Therefore, this part is further divided into zone A and zone B according to the influence of in-situ stress. In Zone A shown in Fig. 9, when L is certain, the number of cracks in the rock mass is more and the damage around the blasthole is more serious. When L = 2 m and L = 3 m, the five working conditions in zone A have better penetration in the crack network diagram. When L = 4 m, the interaction between the hollow hole effect and blasthole exists, but it is weaker compared with L = 2 m and L = 3 m. Therefore, it mainly shows that the damage degree around the blasthole is more serious compared with that of zone B under the same condition. While in zone B when L is certain, the number of cracks and the degree of destruction within the rock body are less and weaker compared to zone A, mainly due to the increasing in-situ stress. From the overall number of cracks and the degree of rock destruction in zone A and zone B, when the distance between blasthole and hollow hole is certain, the overall trend of the number of cracks and the degree of rock destruction decreases and weakens with the increase of in-situ stress, because the interaction between hollow hole effect and blasthole slows down the suppression effect of in-situ stress when the distance between blasthole and hollow hole is smaller, this effect is more obvious, the stronger the effect of overcoming in-situ stress and increasing crack penetration.

## Microscopic analysis of rock blasting

### Comparison of contact force chains

**Figure 10:**
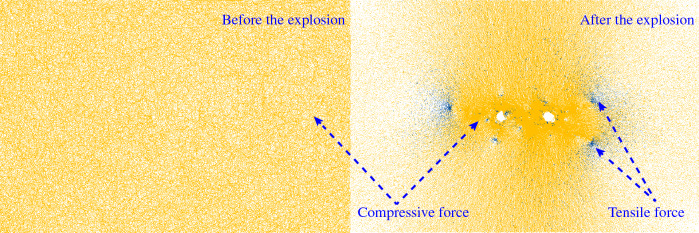
Schematic diagram of contact force chain.

Under blasting load, the magnitude of inter-particle contact force is a key indicator to reflect the degree of inter-particle action. The particle expansion method is a blasting method based on discrete elements, and the inter-particle contact force is generally expressed by means of force chains, and the thickness and color shades of the force chains reflect the magnitude of the inter-particle contact force. In this paper, test with the help of PFC^2D^, from Fig. 10 can be observed before and after the test inter-particle contact force chain, from the comparison of the two figures can be obtained, before the explosion of explosives inter-particle contact force is small (lighter color), uniform distribution; after the explosion of explosives, blasthole near the degree of extrusion is higher, the contact force is larger (darker color), blasthole around the rock body by strong pressure The formation of broken area, the main crack tip area rock body due to the presence of the pull stress play a crack development and extension of the role of the main crack to tension damage (orange indicates compression force, blue indicates tensile force).

### Distribution of contact force chain

From the results of the force chain distribution, it can be seen that during the blasting process, the circumferential tensile force and radial pressure are generated around the blasthole, and the crack is mainly related to the circumferential tensile force. The tension and pressure propagate outward with the stress wave centered on the blasthole. As part of the circumferential tensile force generated by two blastholes propagates to each other, the tensile force converges between the blastholes, making the rock between the blastholes subject to larger tensile forces, and when the tensile force value exceeds the tensile limit of the rock, the force chain breaks and the rock is damaged. If the distance between the two blastholes is far, the tension force between the blastholes is small enough to break the force chain, and the rock will not be damaged and crack will occur. Therefore, adding blasthole will produce stress concentration effect, which will further cause the rock crack.

**Figure 11:**
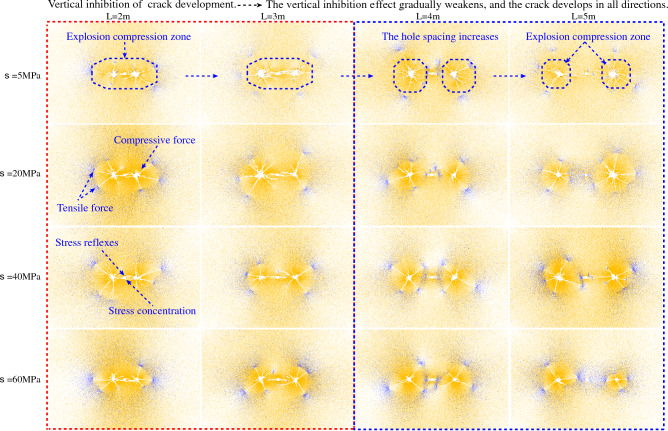
Contact force chain diagram.

As shown in Fig. 11 (5 MPa) the distribution of the force chain at a lower initial stress field. Overall, most of the inter-particle contacts are in a compressed state, indicating that the explosion generates compression waves that cause the particles to squeeze each other. At the same initial stress field ($\sigma$) but different horizontal distance (L) between blasthole and blasthole, the force state of the particles inside the whole rock body also varies greatly.

As shown in Fig. 11, at different in-situ stress, L = 2 m and 3 m, when the distance between blasthole and hollow hole is the smallest, the interaction between blasthole and blasthole is relatively strong, leading to the superposition of stress waves generated by each of the two blastholes inside the rock mass between blasthole, as shown in The force chain in the part of the rock body perpendicular to the blasthole linkage direction (Y direction) near the blasthole is darker than that in other parts, indicating that this part of the rock body is under relatively higher pressure in the Y direction, while the rock body is under higher tension in the X direction, and this phenomenon is closely related to the placement of the blasthole. the higher pressure in the Y direction plays a suppressive role in the development of the crack, while the tension in the X direction plays a suppressive role in the development of the crack. This phenomenon is closely related to the location of the blasthole. The larger pressure in the Y direction inhibits the development of the crack, while the tension in the X direction promotes the development of the crack, which also confirms that the final form of the crack is more developed in the X direction and relatively less developed in the Y direction, and this phenomenon is more related to L.

The Y-directional pressure is relatively less at L = 4 m and 5 m than at L = 2 m and 3 m under different in-situ stress, which leads to a weaker effect of Y-directional crack inhibition and eventually the crack is more developed in all directions. At L = 2 m and 3 m, the force chain around blasthole-hollow-hole-blasthole is the darkest, indicating that the rock is in the high pressure zone and the zone is oval-shaped overall. The part of the rock body outside the contour of the high pressure zone is subject to tensile force, especially the tip part of the main crack. With increasing L, the high pressure zone evolves from an ellipse to two independent circles, and the change of the shape of the high pressure zone can reflect the weakening of the interaction between blasthole as L increases. In addition, the force chain around the hollow hole appears to be strained as L increases, indicating that the distance between blastholes is far, and the tensile force propagating around the hollow hole is not enough to break the force chain and thus crack is generated.

Collectively, it can be seen that when the blasthole spacing is small (less than 2.5 times the radius of the crushing zone), the pressure strain perpendicular to the blasthole linkage direction inhibits the development of crack, and the pull strain in the parallel blasthole linkage direction promotes the development of crack, taking the 2.3 chapter crushing zone radius as the benchmark. When the blasthole spacing was larger, the effect of inhibiting crack was weakened, and crack was more developed in all directions. For the connection between microscopic and microscopic. At the microscopic contact force level, if a common elliptic compression zone is formed near the double hole after the explosive explosion, it indicates that the empty hole is greatly affected by the double explosive load at the same time, the stress concentration near the empty hole is obvious, and the through crack is easy to occur. On the macro level, the hole spacing is reasonable. Therefore, reasonable hole spacing can be selected through the evolution of contact force chain.

### Number of contact force chain

The change of contact force inside the rock body after the explosion of explosives is a key factor affecting the effect of rock breaking. In order to study the internal force changes of the rock body after blasting, this chapter studies the number of contact forces inside the rock body after the explosion of explosives in different working conditions from a fine viewpoint, and the number of contact forces in each direction is counted, and the direction of the internal contact force after the explosion of explosives is analyzed at the level of the number of force chains, and the direction of the contact force under different working conditions is counted and studied.

**Figure 12:**
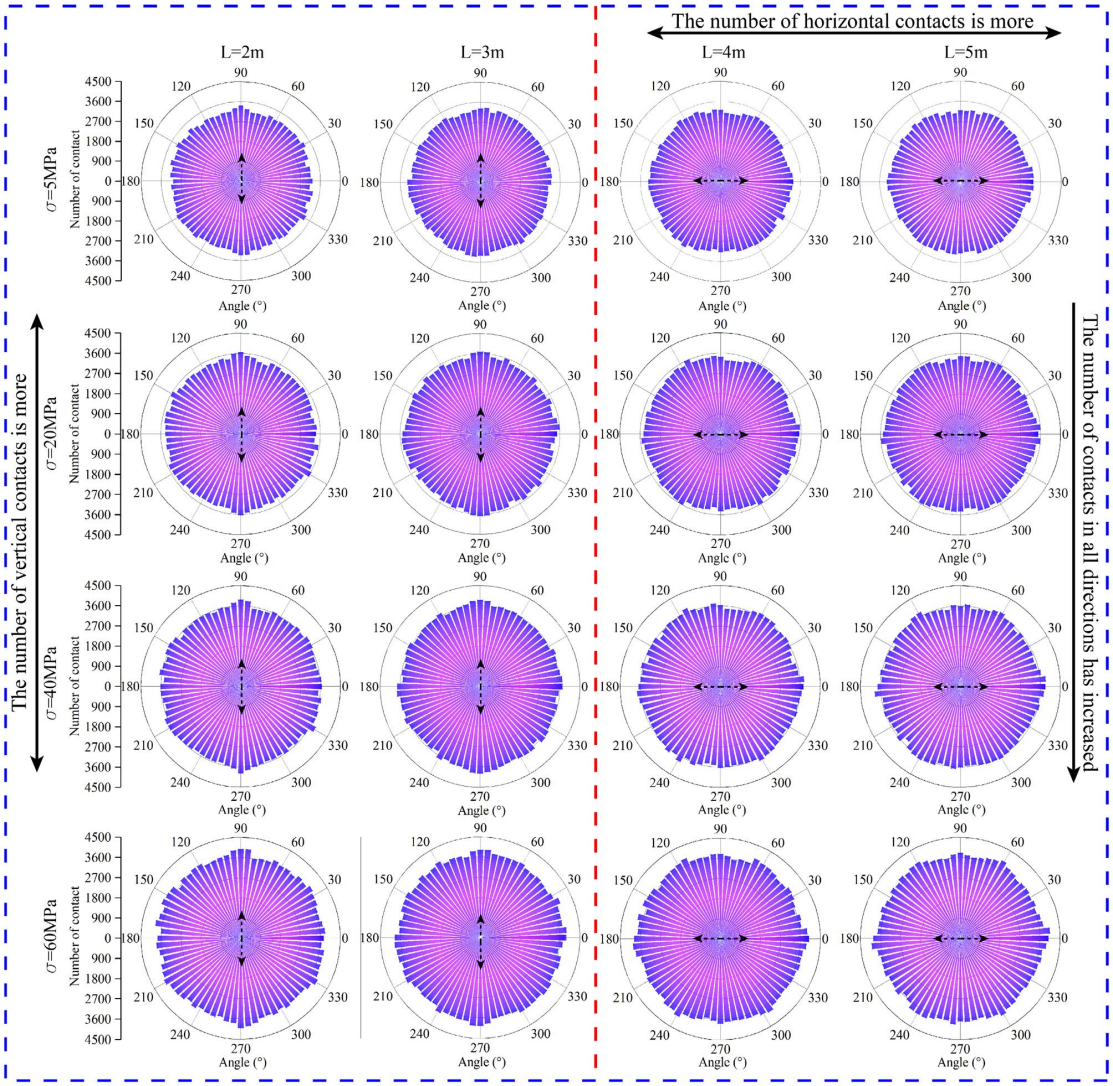
Distribution of number of contact forces and angles.

As shown in Fig. 12, when the distance L between blasthole and blasthole is 2 m and 3 m, the initial stress $\sigma$ is 5 MPa, the number of force chains in each direction is basically the same, and the number of contact force chains in the 90°–270° direction is relatively more (the direction with relatively more contact force chains is called the main force direction in this paper), indicating that the force in the vertical blasthole linkage This is consistent with the results obtained from the distribution of force chains in the former direction. With the increasing of the initial stress $\sigma$, the number of contact force chains in each direction increases uniformly, indicating that when the in-situ stress increases, the internal forces in the rock mass also increase uniformly. When blasting is completed, the number of force chains in the vertical blasthole direction (90°–270°) is higher, indicating that the forces inside the rock mass are greater in the direction of the vertical blasthole line during blasting compared with the other directions, and this rule is consistent for different initial stress conditions at L = 2 m and 3 m.

When $\sigma$ = 5 MPa, the main force direction changes from 90°–270° (L = 2 and 3 m) to 0°-180° (L = 4 and 5 m) as L increases, i.e., mainly from the vertical blasthole line direction to the parallel blasthole line direction. When $\sigma$ keeps changing, the overall law is basically consistent with $\sigma$ = 5 MPa working condition. Comprehensive can be obtained, with 2.3 chapters crushing zone radius as the reference, blasthole and hollow hole distance in less than 2.5 times the radius of the crushing zone, the main force direction perpendicular to the blasthole line direction; with the increase of blasthole spacing, the vertical direction of pressure stress inhibit crack effect is weakened, the main force direction parallel to the blasthole line direction.

For the connection between the number of microscopic and microscopic contact forces. When the crack penetration effect is good, the number of contact forces perpendicular to the hole line is larger. On the contrary, when the effect is poor, there are more contacts parallel to the direction of the gun hole line.

## Analysis of peak stress near the empty hole

The use of guide hole rock pretreatment method is a method to increase the free surface near the blasthole, the presence of free surface after the explosion of explosives can lead to stress reflection, transmission, stress concentration and other phenomena, in order to more clearly observe the changes in the maximum stress near the hollow hole, analyze the influence of the guide hole on the explosion stress, this paper sets up a total of four measurement circles near the hollow hole for real-time detection of the maximum stress near the hollow hole.

### Setting of the measurement circle

**Figure 13:**
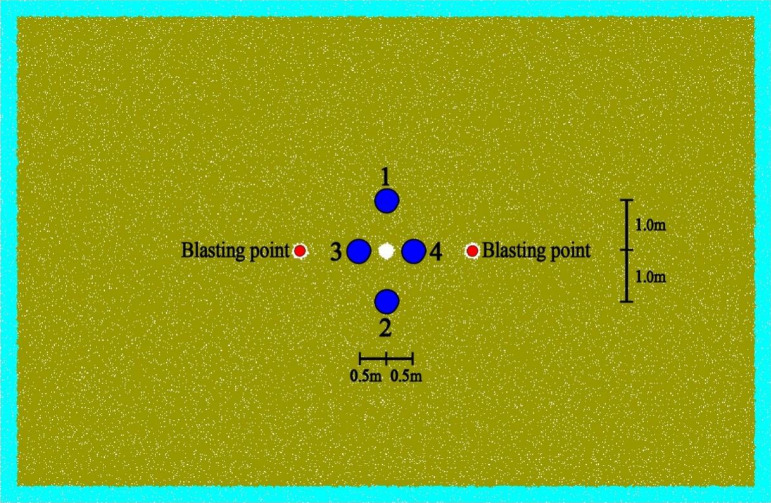
Schematic diagram of measurement circles.

When the distance L is the same between the two blast points, in order to study the variation law of peak stress near the hollow hole under different in-situ stress, four measuring circles are set near the hollow hole for the detection of peak stress in this paper. Among them, two measurement circles are set in the vertical direction, numbered 1 and 2, measurement circle No. 1 above the hollow hole at 1 m from the hollow hole, measurement circle No. 2 below the hollow hole at 1 m from the hollow hole, measurement circles No. 1 and 2 mainly detect the vertical peak stress near the hollow hole; two measurement circles are set in the horizontal direction, numbered 3 and 4, measurement circle No. 3 in the left direction of the hollow hole at 0.5 m from the hollow hole, measurement circle No. 4 in the right The specific distribution is shown in Fig. 13.

### Analysis of vertical peak stress

**Figure 14:**
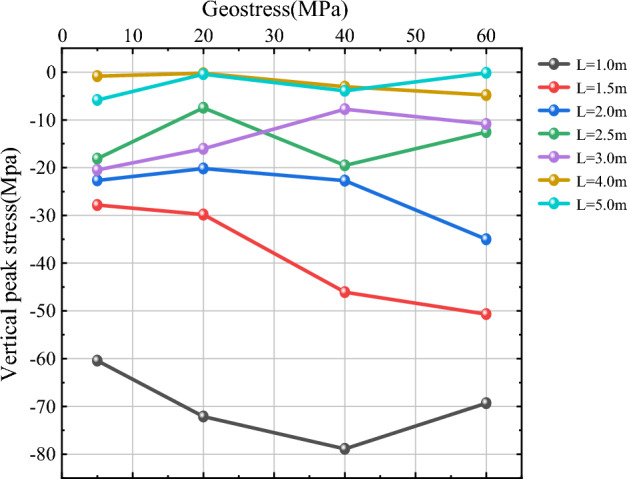
Vertical peak stress curve of measurement circle 1.

**Figure 15:**
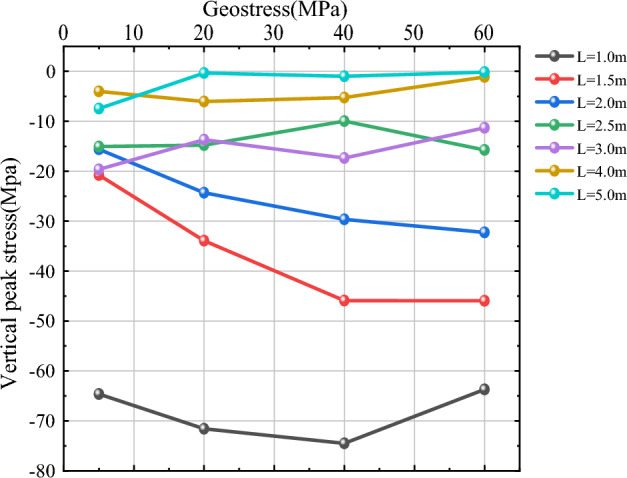
Vertical peak stress curve of measurement circle 2.

As shown in Figs. 14 and 15, in order to study more clearly the effect of in-situ stress $\sigma$ on vertical peak stress near the hollow hole at different blast point distances L, three more working conditions of L = 1 m, 1.5 m and 2.5 m are added in this section based on L = 2 m, 3 m, 4 m and 5 m. For the measurement points 1 and 2, when L is small (L = 1 m, 1.5 m, 2 m), the value of vertical peak stress near the hollow hole is larger and more variable as the in-situ stress increases; when L = 1 m, the vertical peak stress at the measurement circle of 1 and 2 both show the trend of increasing first and then decreasing, and the vertical peak stress reaches the maximum at $\sigma$ = 40 MPa; when L = 1.5 m and 2 m, the vertical peak stress above and below the hollow hole shows a trend of gradually increasing with the increase of in-situ stress. When L is larger (L = 2.5 m, 3 m, 4 m, 5 m), the vertical peak stress near the hollow hole is basically unaffected by in-situ stress as the in-situ stress increases; when L = 2.5 m and 3 m, the vertical peak stress fluctuates around a specific value; when L = 4 m and 5 m, the vertical peak stress basically remains around a specific value and is less affected by The influence of in-situ stress is less. Comprehensive can be obtained, continue to 2.3 chapter crushing zone radius as the benchmark, 2.0 times the radius of the crushing zone as the dividing point, when the distance L between the blast point is small, in-situ stress has a greater impact on the vertical peak stress near the hollow hole, the vertical peak stress largely increases with the increase of in-situ stress; When L is larger, the influence of in-situ stress on vertical peak stress becomes smaller and smaller, and the value of vertical peak stress is basically maintained around a specific value.

### Analysis of horizontal peak stress

**Figure 16:**
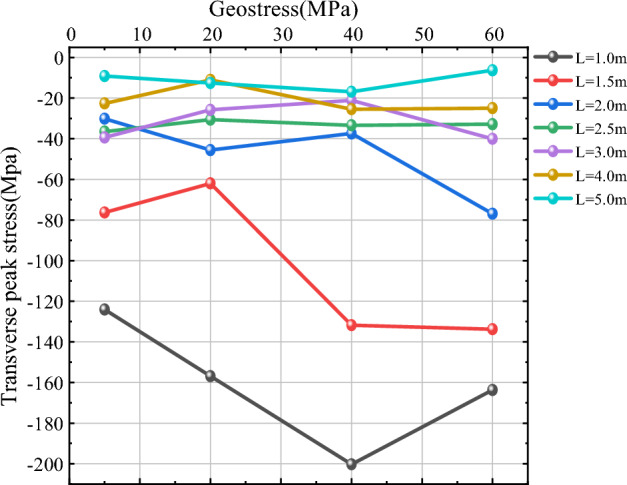
Transverse peak stress curve of measurement circle 3.

**Figure 17:**
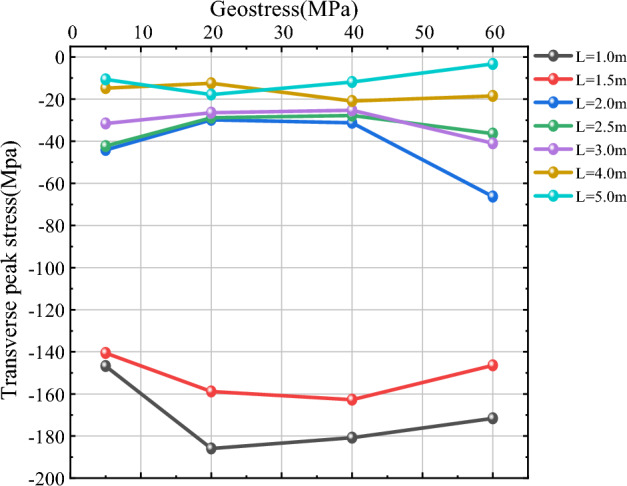
Transverse peak stress curve of measurement circle 4.

As shown in Figs. 16 and 17, for the level of transverse peak stress near the hollow hole, when L is small (L = 1 m, 1.5 m, 2 m), with the increase of in-situ stress, the value of transverse peak stress near the hollow hole is larger and changes more, when L = 1 m and 1.5 m, the vertical peak stress at the measurement circle of No. 3 and No. 4 both show a trend of increasing first and then decreasing, and the left side near the hollow hole (No. 3), the change in transverse peak stress is larger, and the change in the right side (No. 4) is smaller; when L = 2.0 m, the transverse peak stress at the measurement circle of No. 3 and No. 4 shows a trend of firstly increasing smoothly and then increasing, and when $\sigma$ < 40 MPa, the transverse peak stress shows a basic smooth state, and when 40 MPa < $\sigma$ < 60 MPa, the transverse peak stress shows a gradually increasing state. When L is larger (L = 2.5 m, 3 m, 4 m, 5 m), with the increase of in-situ stress, the transverse peak stress near the hollow hole is little affected by the in-situ stress, and the transverse peak stress is basically maintained at a specific value. Comprehensive can be seen, with 2.0 times the radius of the crushing area as the dividing point, when the distance L between the blowing points is small, the transverse peak stress near the hollow hole is more sensitive to in-situ stress, when the distance L between the blowing points is large, the influence of in-situ stress on the transverse peak stress is small, and the value of transverse peak stress is basically maintained at a specific value or so.

## Conclusion

In this paper, using the particle expansion discrete element blasting simulation method^25^, based on the rock body containing a guide hole, the influence of blasthole spacing and in-situ stress on the blasting effect is considered, and the effect of crack penetration effect, particle contact, near the hollow hole is analyzed when the blasthole spacing L and in-situ stress $\sigma$ are varied. The variation law of stress. The main conclusions are as follows:

(i) The increase of hollow holes between blastholes can enhance the crack penetration effect between blastholes, and the penetration effect is more obvious when the distance between blasthole and hollow holes (half of blasthole spacing L) is less than 2.5 times the radius of crushing zone, and outside this range, the penetration effect is obviously weakened or even disappeared.
(ii) At the level of contact force chain, when the distance between blasthole and hollow hole is less than 2.5 times the radius of crushing zone, the rock around blasthole-hollow hole-blasthole is in a high pressure zone similar to ellipse, the pressure in the direction perpendicular to the blasthole linkage is higher, the tension in the direction parallel to the blasthole is higher, and the pressure in the direction perpendicular to the blasthole The pressure in the direction of vertical blasthole linkage inhibits the development of crack, and the tension in the direction parallel to the blasthole linkage promotes the development of crack. With the increase of L, the pressure in the direction of straight blasthole linkage gradually decreases, and the effect of inhibiting cracks weakens, and the high pressure zone evolves from an ellipse to two independent circles, and cracks begin to develop in all directions.
(iii) For the contact number level, when the distance between the blasthole and the hollow hole is less than 2.5 times the radius of the crushing zone, there are more contact force chains in the direction perpendicular to the blasthole line at different in-situ stress, indicating that the force in the direction of the vertical blasthole line is larger; with the increase of L, the main force direction at different in-situ stress changes from As L increases, the main force direction changes from the vertical blasthole line direction to the parallel blasthole line direction at different in-situ stress, and the force in the horizontal direction is greater; when L is constant, the number of contact forces increases uniformly with the increase of in-situ stress, and the force on the rock body also increases uniformly.
(iv) When the distance between the blast points L is small, the transverse and vertical stresses near the hollow hole are more sensitive to in-situ stress, when the distance between the blast points L is large, the influence of in-situ stress on the transverse and vertical stresses near the hollow hole is smaller, and the values of transverse and vertical stresses are basically maintained at The values of lateral and vertical stresses are basically maintained around a specific value.

## Data Availability

The datasets used and/or analysed during the current study available from the corresponding author on reason.
